# Systemic lipopolysaccharide-mediated alteration of cortical neuromodulation involves increases in monoamine oxidase-A and acetylcholinesterase activity

**DOI:** 10.1186/s12974-015-0259-y

**Published:** 2015-02-25

**Authors:** Zhi Ming, Caitlin A Wotton, Robert T Appleton, John C Ching, Matthew E Loewen, Grzegorz Sawicki, Lane K Bekar

**Affiliations:** Department of Pharmacology, University of Saskatchewan, 107 Wiggins road, Saskatoon, SK S7N 5E5 Canada; Veterinary Biomedical Sciences, University of Saskatchewan, 52 Campus Drive, Saskatoon, SK S7N 5E5 Canada; Department of Clinical Chemistry, Medical University of Wroclaw, Wybrzeże L. Pasteura 1, Wroclaw, 50-556 Poland

**Keywords:** Inflammation, Phasic neuromodulator release, Cortical network, Cortical inhibition

## Abstract

**Background:**

Lipopolysaccharide (LPS)-mediated sickness behaviour is known to be a result of increased inflammatory cytokines in the brain. Inflammatory cytokines have been shown to mediate increases in brain excitation by loss of GABA_A_-mediated inhibition through receptor internalization or inactivation. Inflammatory pathways, reactive oxygen species and stress are also known to increase monoamine oxidase-A (MAO-A) and acetylcholinesterase (ACh-E) activity. Given that neuromodulator actions on neural circuits largely depend on inhibitory pathways and are sensitive to alteration in corresponding catalytic enzyme activities, we assessed the impact of systemic LPS on neuromodulator-mediated shaping of a simple cortical network.

**Methods:**

Extracellular field recordings of evoked postsynaptic potentials in adult mouse somatosensory cortical slices were used to evaluate effects of a single systemic LPS challenge on neuromodulator function 1 week later. Neuromodulators were administered transiently as a bolus (100 μl) to the bath perfusate immediately upstream of the recording site to mimic phasic release of neuromodulators and enable assessment of response temporal dynamics.

**Results:**

Systemic LPS administration resulted in loss of both spontaneous and evoked inhibition as well as alterations in the temporal dynamics of neuromodulator effects on a paired-pulse paradigm. The effects on neuromodulator temporal dynamics were sensitive to the Monoamine oxidase-A (MAO-A) antagonist clorgyline (for norepinephrine and serotonin) and the ACh-E inhibitor donepezil (for acetylcholine). This is consistent with significant increases in total MAO and ACh-E activity found in hemi-brain samples from the LPS-treated group, supporting the notion that systemic LPS administration may lead to longer-lasting changes in inhibitory network function and enzyme (MAO/ACh-E) activity responsible for reduced neuromodulator actions.

**Conclusions:**

Given the significant role of neuromodulators in behavioural state and cognitive processes, it is possible that an inflammatory-mediated change in neuromodulator action plays a role in LPS-induced cognitive effects and could help define the link between infection and neuropsychiatric/degenerative conditions.

## Introduction

Peripheral lipopolysaccharide (LPS) injection is widely known to induce sickness behaviour [1-6], mediated primarily via the inflammatory cytokines IL-1β [6], IL-6 [7] and TNF-α [6]. Transduction of this peripheral response into the CNS is accomplished via indirect stimulation of vagal sensory nerve activity [8,9] as well as more direct effects transduced through endothelial prostaglandin synthesis [2], activation of macrophage-like cells in the circumventricular organs, and direct transduction across the blood brain barrier [10] to activate local microglial cells [11]. All pathways lead to elevated prostaglandins and inflammatory cytokines in the brain that are thought to mediate the various sickness behaviours that include fever, loss of activity, loss of appetite, impaired cognition, anxiety and depression [9,12,13]. The systemic inflammatory response subsides within 7 days [11], sickness behaviour by 3 days [13], but CNS inflammation and impact may be long-lasting and potentially involved in neurodegenerative processes. Although cytokines are known to be responsible for sickness behaviour, the mechanisms by which they alter brain function are still largely unknown.

Regulation of CNS inhibition seems to be one mechanism by which cytokines alter brain state. Interleukin-6 has been shown to lead to a decrease in the inhibition/excitation ratio in the rat temporal cortex that was postulated to contribute to a hyper-excitable state associated with various neurological or psychiatric conditions [14]. Likewise, TNF-α, implicated in plasticity and synaptic scaling [15,16], can also decrease the inhibition/excitation ratio via increasing glutamate AMPA receptor insertion and GABA_A_ receptor internalization in central synapses [17]. Thus, inflammatory cytokines are widely associated with loss of CNS inhibition.

The enzymes responsible for breakdown/inactivation of many neuromodulators are sensitive to stress and inflammation. Monoamine oxidase-A (MAO-A; breakdown norepinephrine and serotonin) expression has been widely shown to increase in response to chronic stress [18], glucocorticoids [19,20] and inflammatory p38 MAPK activity [21]. Acetylcholinesterase (ACh-E; breakdown acetylcholine) expression has been shown to increase in response to IL-1 [22] and oxidative stress [23,24]. Given the effects of inflammation on MAO-A and ACh-E, the purpose of this study was to assess the impact of systemic LPS-mediated neuroinflammation on neuromodulator regulation of somatosensory cortical networks. Results showed that a simple extracellular field recording paradigm can be used to measure functional neuromodulator alterations following an inflammatory event. We show that alterations in neuromodulator shaping of cortical networks 7 days following systemic LPS administration involve changes in MAO-A and ACh-E activity. Given the impact that neuromodulators have on determining behavioural state, our results support the notion that inflammatory changes in neuromodulator action may be involved in cognitive and emotional aspects of neuropsychiatric or neurodegenerative processes.

### Experimental procedures

#### Animal treatment

Male C57Bl6 mice were purchased from Charles River Laboratories (Charles River Laboratories Inc, Quebec, Canada) and allowed to adjust to new environment for a minimum of 1 week before experimentation. Mice were individually housed for 1 week after injection and provided food and water *ad libitum* in a colony room maintained at 20°C with a 12:12-h light-dark cycle (lights on at 8 a.m.). Experiments were in accordance with the guidelines of the Canadian Council on Animal Care and approved by the University of Saskatchewan Committee on Animal Care and Supply.

Mice (8 to 10 weeks old) received a single intraperitoneal injection of either vehicle (0.9% saline) or lipopolysaccharide (strain 0111:B4, >500,000 endotoxin units/mg; 3 mg/kg). One week following a single injection, animals were sacrificed for experiments. We chose to assess neuromodulator effects 1 week after injection when typical sickness behaviours have subsided to assess longer-lasting effects that may be linked to neuropsychiatric or neurodegenerative processes.

### Brain slice preparation

**Figure 1:**
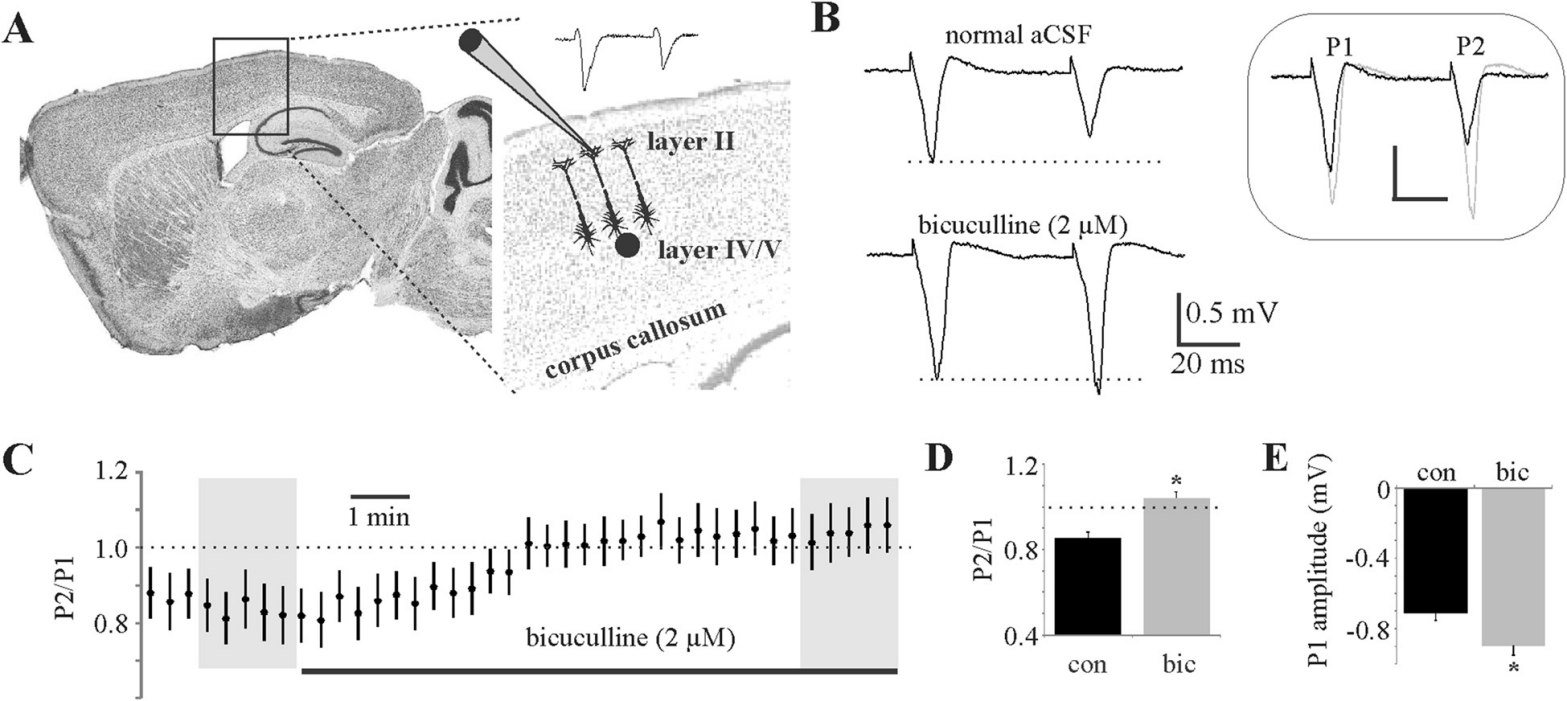
**Cortical paired-pulse suppression is dependent on inhibitory GABA**_**A**_ **activity. (A)** Schematic of a sagittal brain slice illustrating the recording and stimulating electrode placements for the model used in these studies. **(B)** Representative samples of paired-pulse fPSP recordings in layer II/III before and after bicuculline addition to the slice perfusate. **(C)** A time series (recordings every 20 s) representation of the average ratios of pulse 2 amplitude over pulse 1 amplitude before and during bicuculline addition to the bath perfusate. **(D)** P2/P1 ratio comparison before and after bicuculline addition for assessment of evoked inhibition. **(E)** P1 amplitude comparison before and after bicuculline for assessment of spontaneous inhibition. For D and E, the average of five responses were obtained from each recording at time points demarcated by grey shading in C. *N* = 17 slices from five mice. *P < 0.001, paired *t*-test. Con, control; bic, bicuculline; P1, pulse 1; P2, pulse 2; aCSF, artificial cerebral spinal fluid; min, minutes.

The brain was rapidly removed on ice and submersed in ice-cold artificial cerebral spinal fluid (aCSF) containing (mM): NaCl 123, KCl 3.5, NaH_2_PO_4_ 1.2, CaCl_2_ 2, MgSO_4_ 10, NaHCO_3_ 26, ascorbic acid 0.04, dextrose 10 and kynurenic acid 1. Kynurenic acid and high magnesium are used to block glutamatergic-mediated excitotoxicity. The brain was cut down the midline, and half of the brain was glued midline face down on a brain chuck for placement on the vibratome (Leica VT 1200, Leica Biosystems, Wetzlar, Germany). Three to five 350-μm thick sagittal slices were obtained through hindlimb/forelimb somatosensory cortex (Figure 1A). Somatosensory cortex was chosen as representative for neuromodulator action on sensory processing. Slices for electrophysiology were immediately transferred to a recovery chamber at 30°C (1 h; thereafter room temperature) in normal aCSF containing (mM): NaCl 123, KCl 3.5, NaH_2_PO_4_ 1.2, CaCl_2_ 2, MgSO_4_ 2, NaHCO_3_ 26, ascorbic acid 0.04 and dextrose 10.

### Electrophysiology

Extracellular electrophysiological recordings were carried out on C57Bl6 mouse brain slices at room temperature. Slices were placed in a perfusion chamber (approximately 2 ml volume) and perfused (approximately 4 ml/min) with normal aCSF gassed with 5% CO_2_ and 95% O_2_ and imaged using a Nikon SMZ1000 stereozoom microscope (Nikon Corporation, Chiyoda, Tokyo, Japan) for placement of recording and stimulating electrodes. Recordings were obtained with a differential amplifier (DP311; Warner Instruments, Hamden, CT, USA) connected to a Digidata 1440A (Molecular Devices, Sunnyvale, CA, USA) using PClamp 10.2 software (Molecular Devices, Sunnyvale, CA, USA). Signals were captured at 2 kHz, high-pass filtered at 1 Hz and low-pass filtered at 1 kHz. Recording electrodes (filament thin-wall glass; WPI, Sarasota, FL, USA) were pulled on a vertical Narishige PC-10 two-step puller (Narishige, Tokyo, Japan), filled with 0.9% saline (resistance <4 MΩ) and positioned in layer II/III of hindlimb somatosensory cortex (Figure 1A). Stimulation (15 to 30 μA; 0.5 ms; 0.33 Hz) was applied to layer IV/V (Figure 1A) using a concentric bipolar-stimulating electrode (CBARC75, FHC Inc, Bowdoin, ME, USA) via a constant current stimulator (Iso-Flex; A.M.P.I., Jerusalem, Israel) controlled by PClamp through the digital output on the Digidata 1440A (Molecular Devices, Sunnyvale, CA, USA). Only recordings (approximately 60% of max) of maximal postsynaptic potentials (PSP) greater than 1.2 mV were used in these studies.

Neuromodulators were delivered to the chamber as a bolus injection (100 μl; Hamilton syringe) directly upstream (approximately 1 mm) of the recording electrode through PE-10 tubing (Harvard Apparatus, Holliston, MA, USA) positioned in the chamber using a bent 16-gauge needle. Unlike the slow and stable increase in neuromodulator concentration seen with addition to the bath perfusate (tonic release associated with level of arousal), bolus application resembles more closely the acute physiological release (phasic release) of neuromodulators associated with environmental stimuli that are necessary for transient modulation of executive functions. Furthermore, bolus delivery enables evaluation and comparison of the temporal profiles of neuromodulator actions. Acetylcholine (ACh; 10 and 5 mM), norepinephrine (NE; 5 and 2 mM) and serotonin (5-HT; 2 and 1 mM) were dissolved in normal aCSF and administered via Hamilton syringe. Given the 2-ml volume of the chamber, a flow rate of approximately 4 ml/min and the necessity of the neuromodulator to diffuse greater than 100 μm into the tissue slice, we guestimate the concentration of neuromodulator to only reach 100 to 1,000-fold lower at the recording site (<50 μM), which is consistent with prior studies assessing neuromodulator function via perfusion delivery [25,26]. Clorgyline (5 μM), donepezil (100 nM) and bicuculline (2 μM) were added to normal aCSF and delivered via the bath perfusate (and slice incubation bath in the case of clorgyline). All chemicals were obtained from Sigma-Aldrich (Sigma-Aldrich, St. Louis, MO, USA), unless otherwise stated.

### Tail suspension test

Mice were suspended by the tail (tape approximately 2 cm from tip of tail) to a surface 50 cm above the table and videotaped for 6 min. Individual videos were manually scored for total immobility time over the 6 min and latency to first immobility using a stop watch by an observer blinded to the treatment group [13,27,28].

### MAO, ACh-E and NO assays

Total brain monoamine oxidase activity was measured using the MAO-Glo™ assay kit (Promega, Madison, WI, USA, Cat.# V1401) that uses a derivative of beetle luciferin as a luminogenic MAO substrate. Mouse hemi-brain tissue was homogenized in cold phosphate buffer 0.1 M (pH 8.0; 4 μl/mg tissue) and protein content measured by the Bradford assay (Bio-Rad Laboratories Inc, Hercules, CA, USA). Brain samples (40 μg protein) were run in triplicates and added to a reaction mixture containing the 4× MAO substrate and reaction buffer provided in the kit as directed. The resulting luminescent signal was read on a luminometer (SpectraMax® M5 Multi-Mode microplate reader, Molecular Devices, Sunnyvale, CA, USA) and all data normalized to the average of the saline-treated group for direct comparison.

Acetylcholinesterase activity was measured using the method described by George Ellman and colleagues in 1961 [29]. Mouse hemi-brain tissue was homogenized in cold phosphate buffer 0.1 M (pH 8.0; 4 μl/mg tissue) and protein content measured by the Bradford assay (Bio-Rad Laboratories Inc, Hercules, CA, USA). Brain samples (20 μg protein) were run in triplicates and added to a reaction mixture containing 300 μl of 0.1 M phosphate buffer (pH 8.0), 2 μl of the substrate 0.075 M acetylthiocholine iodide and 10 μl of 0.01 M 5-5 dithiobis (2-nitrobenzoic acid) (DTNB). Samples were read using a spectrophotometer (Bio-Rad Laboratories Inc, Hercules, CA, USA) at 415 nm in 5-min intervals for 30 min. The maximum slope over a 10-min period was used for analysis. All samples were normalized to the average slope of the saline-treated group for direct comparison.

Nitric oxide was measured using the colorimetric nitric oxide assay Kit (ab65328; Abcam®, Cambridge, UK) that uses Griess reagents to convert nitrite to a deep purple azo compound. Mouse hemi-brain tissue was homogenized in cold phosphate buffer 0.1 M (pH 8.0; 4 μl/mg tissue) and protein content measured by the Bradford assay (Bio-Rad Laboratories Inc, Hercules, CA, USA). Brain samples (60 μg protein) were run in duplicates as directed in the kit, and the absorbance was read at 540 nm using a spectrophotometer (Bio-Rad Laboratories Inc, Hercules, CA, USA).

### Statistics

The data are expressed as mean ± SEM. Statistical significance was assessed using paired or unpaired *t*-tests or a two-way analysis of variance followed by a Scheffé’s multiple comparison method (OpenStat software). Significance was determined when *P* < 0.05.

## Results

### Cortical paired-pulse suppression is dependent on inhibitory GABA_A_ activity

Perfusate application of the GABA_A_ inhibitor bicuculline to cortical slices enables rapid evaluation of spontaneous and evoked inhibitory activity. To assess inhibitory involvement in cortical circuits, we used a paired-pulse paradigm to assess evoked field postsynaptic potentials (fPSP) in layer II/III of hindlimb/forelimb somatosensory cortex (Figure 1A). Although we evoke both field potentials, the first potential is essentially devoid of evoked inhibitory input as inhibitory interneurons are recruited with the first pulse to have impact on the second potential only. Thus, the first pulse of the pair represents spontaneous activity (background inhibition) only, whereas the ratio represents evoked inhibition. Two stimulations (50 ms apart) of layer IV/V with a concentric bipolar electrode typically showed 20% to 40% suppression of the second fPSP that could be abolished by washing the GABA_A_ antagonist bicuculline (2 μM) over the slice in the perfusate (Figure 1B,C,D). This bicuculline-sensitive component of the paired-pulse ratio (Figure 1D), thus, can serve as an indication of the magnitude of cortical evoked inhibition. The corresponding increase in fPSP amplitude with the addition of bicuculline to the perfusate can, likewise, be used as an indication of the level of spontaneous inhibition in the acutely isolated brain slices used in this study (Figure 1E).

### Neuromodulators differentially affect cortical paired-pulse suppression

**Figure 2:**
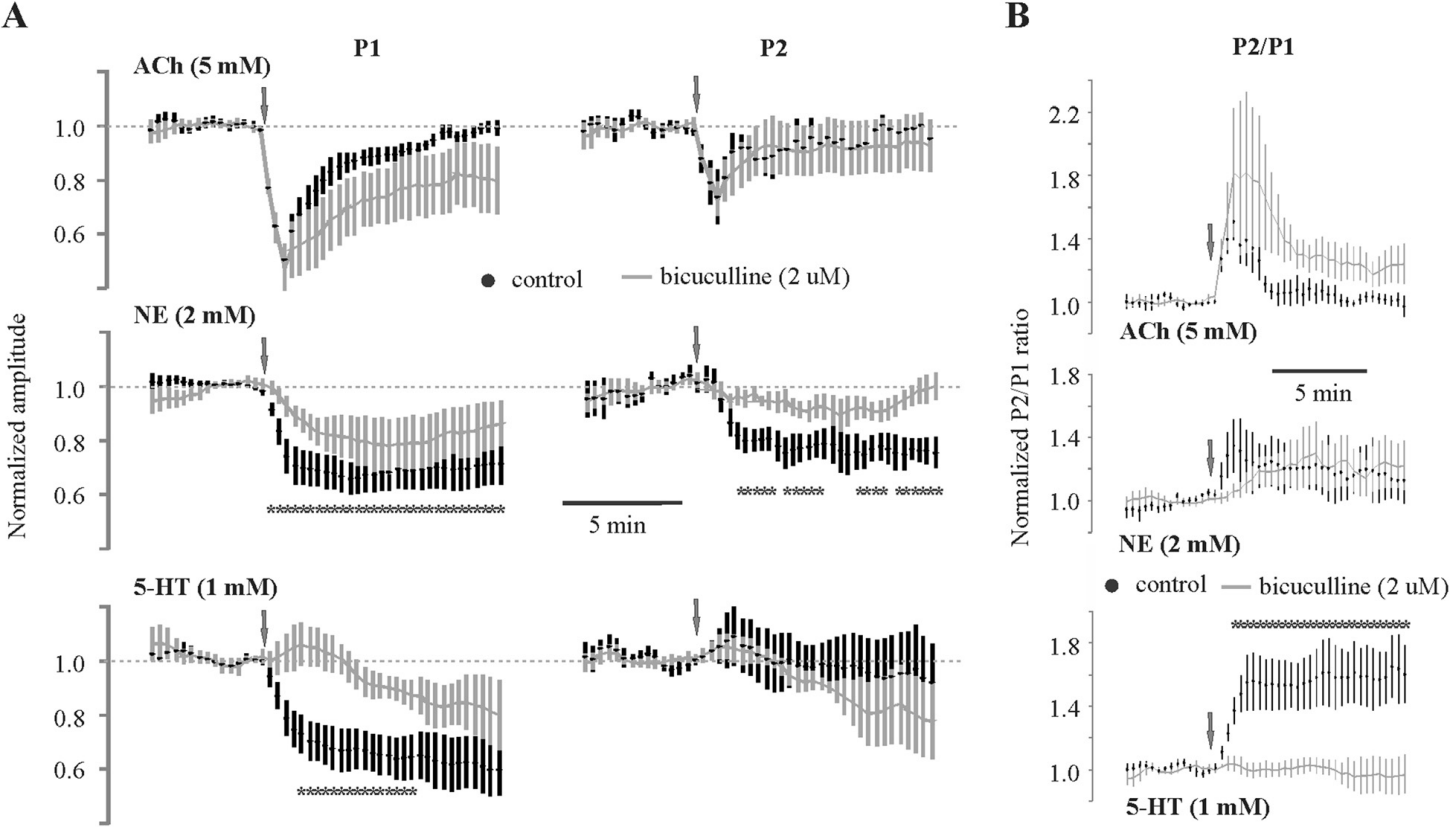
**Neuromodulators recruit different mechanisms to achieve their effects. (A)** Time series recordings (every 20 s) of P1 and P2 amplitude responses to select neuromodulators before and after perfusion with bicuculline for assessment of effects on spontaneous and evoked inhibition. **(B)** Time series recording of P2/P1 ratio before and after bicuculline perfusion for assessment of inhibition on frequency-dependent transmission. Amplitudes or ratios were normalized to the average of ten traces immediately preceding neuromodulator application to enable comparison. *P < 0.05, paired t-test. 5-HT, serotonin (*N* = 5 slices from five mice); ACh, acetylcholine (*N* = 6 slices from five animals); NE, norepinephrine (*N* = 7 slices from four animals); min, minutes; P1, pulse 1; P2, pulse 2.

In these studies, we used a novel and simple combination of local bolus neuromodulator application, GABA_A_ pharmacology and recordings of paired-pulse extracellular fPSPs to evaluate the temporal profiles of ACh, NE and 5-HT effects on inhibitory and excitatory cortical network components. Although all three neuromodulators reduce the amplitude of the first pulse (49.9 ± 4.97% for ACh; 34.6 ± 5.75% for NE; 34.1 ± 7.04% for 5-HT, Figure 2A), only 5-HT and NE effects appear to be sensitive to the GABA_A_ antagonist bicuculline (Figure 2A). We did not observe a bicuculline effect on the ACh-mediated reduction in the first pulse amplitude of the paired-pulse regimen. Serotonin showed the most pronounced dependence on GABA networks in the short term with a much slower GABA_A_-independent suppression developing even after washout (Figure 2A, bottom left).

Acetylcholine and 5-HT show no GABA_A_ involvement in effects on the second pulse, whereas NE-mediated suppression of P2 is significantly reduced following treatment with bicuculline (Figure 2A, right). Assessment of the normalized paired-pulse ratio with and without bicuculline in the perfusate illustrates the dependence of GABA_A_ receptors in the neuromodulator effects on frequency transmission in cortical networks (Figure 2B). Note that effects of 5-HT on the paired-pulse ratio (frequency transmission) are completely blocked by inhibition of GABA_A_ receptors whereas the effects on ACh and NE are more subtle.

### Systemic LPS induces transient behavioural changes and an increase in nitric oxide production

**Figure 3:**
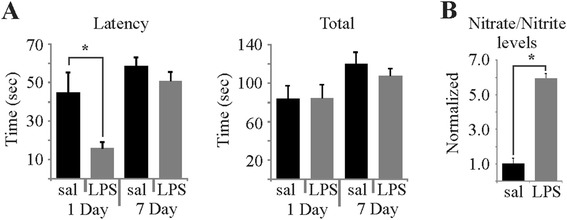
**Systemic LPS induces transient effects in the tail suspension test and an increase in nitric oxide production. (A)** Histograms comparing the latency to the first immobility and total immobility time at 1 day (*N* = 10, *P < 0.05, unpaired *t*-test) versus 7 days (*N* = 8, unpaired *t*-test) post LPS treatment. **(B)** Histogram showing nitrate/nitrite levels of hemi-brain homogenates collected 7 days post LPS treatment (*N* = 9 per group; *P < 0.001, unpaired *t*-test) LPS, lipopolysaccharide.

Verification that systemic LPS had a behavioural effect on the mice was accomplished by performing the tail suspension test (a simple behavioural test of despair) in separate cohorts of animals 1 day (*N* = 10 per group) and 7 days (*n* = 8 per group) after systemic LPS administration. Animals were assessed for total immobility and latency to first immobility during 6 min of tail suspension. Latency to first immobility was significantly lower in LPS-treated animals 1 day after LPS but not 7 days after (Figure 3A, left). Interestingly, no difference was observed in total immobility at either day after LPS treatment (Figure 3A, right).

As the inducible form of nitric oxide synthase (iNOS) is intimately involved in the immune inflammatory response [30,31], we also performed a simple assay for levels of nitrates/nitrites in brain homogenates as a measure of nitric oxide production (NO rapidly oxidized to nitrite and nitrate). Despite not seeing any effect in the tail suspension test at 7 days post LPS, the nitric oxide assay showed a sixfold increase in nitric oxide production in LPS-treated animals compared to saline-treated controls (Figure 3B), indicative of a neuroinflammatory effect.

### Systemic LPS reduces both cortical inhibition and neuromodulation

**Figure 4:**
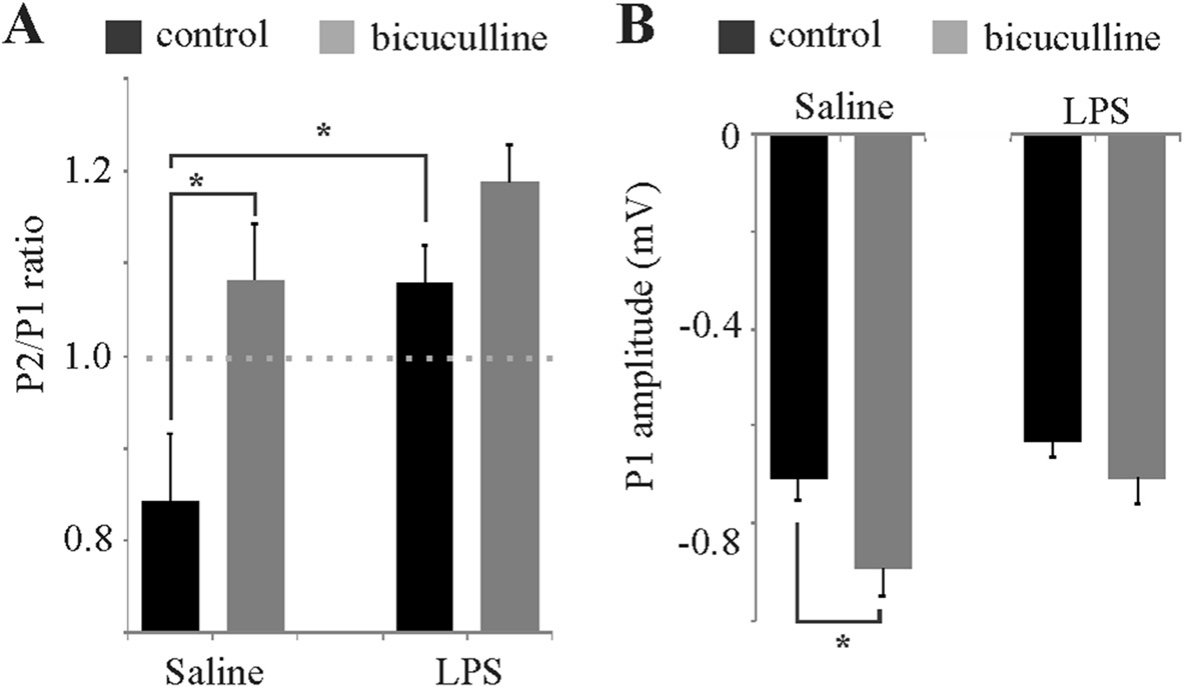
**LPS reduces both spontaneous and evoked cortical inhibition. (A)** Histograms comparing the bicuculline sensitivity of the paired-pulse ratios (*P < 0.05, Scheffé’s multiple comparison). **(B)** Comparison of the bicuculline sensitivity of the first pulse amplitude between saline- and LPS-treated groups (*N* = 18 slices from five animals in each group; *P < 0.001, paired *t*-test). LPS, lipopolysaccharide; P1, pulse 1; P2, pulse 2.

Perfusion of the tissue slice with the GABA_A_ antagonist bicuculline while monitoring the extracellular fPSPs (0.33 Hz) demonstrates a loss of the GABA_A_-dependent effects on P1 and P2 in slices from animals pretreated with a single LPS injection 1 week earlier. The average of five paired-pulse recordings was collected from each slice before and after perfusion with bicuculline for comparison of ratios and bicuculline-sensitive changes in P1 amplitude (Figure 4). The P2/P1 ratio after LPS treatment was significantly larger than that of the control as well as showing a loss of bicuculline sensitivity (Figure 4A). Likewise, whereas P1 amplitude showed a significant increase with bicuculline in control slices, LPS-treated slices showed no P1 amplitude increase in response to bicuculline addition (Figure 4B). Thus, LPS treatment significantly reduces both spontaneous (P1) and evoked (ratio) inhibition.

**Figure 5:**
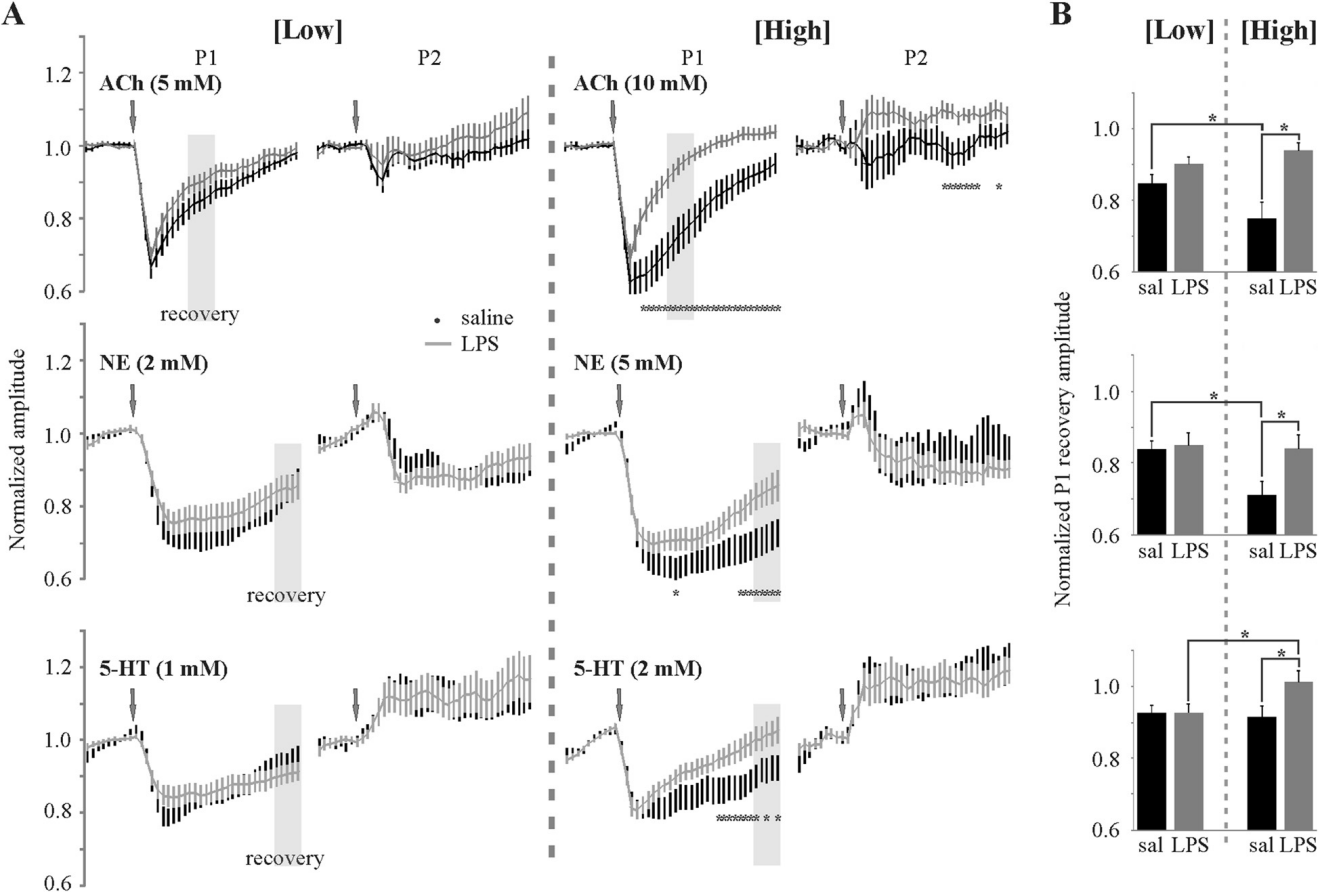
**LPS alters neuromodulator action on cortical networks. (A)** Low and high concentrations of bolus neuromodulator application demonstrate an LPS-mediated concentration-dependent increase in the rate of response recovery. (Minimum of 24 slices from eight animals in each group; *P < 0.05, unpaired *t*-test) **(B)** Histograms comparing the normalized recovery of the first pulse as determined by the average of five stimulations demarcated by the grey-shaded boxes in **(A)** (*P < 0.05, Scheffé’s multiple comparison) ACh, acetylcholine; NE, norepinephrine; 5-HT, serotonin; LPS, lipopolysaccharide; P1, pulse 1; P2, pulse 2.

In addition to effects on cortical inhibition, systemic injection of LPS also results in an altered neuromodulator effect on cortical networks. Experiments were performed with two different bolus concentrations each of ACh, NE and 5-HT to ensure we were getting adequate action on the various receptor subtypes (neuromodulator bolus is diluted substantially before reaching the recording site deep in the slice) (Figure 5). Initial experiments with the higher neuromodulator concentrations demonstrated a significant LPS-induced change in the temporal recovery of the neuromodulator effects on the first pulse of the paired-pulse regimen (Figure 5A, right) with minor effects on the second pulse only seen with ACh (Figure 5A, right). Subsequent use of lower neuromodulator concentrations was unable to replicate the LPS-mediated changes to neuromodulator actions (Figure 5A, left). Additional lowering of neuromodulator concentrations resulted in many recordings not showing a neuromodulator response (data not shown). Combined, these results demonstrate a significant neuromodulator concentration-dependent effect on the temporal neuromodulator recovery dynamics with LPS treatment (two-way ANOVA; Figure 5B). A significant increase in recovery of the neuromodulator effect is seen in LPS-treated animals for all neuromodulators examined (Figure 5B, high concentration).

### LPS-mediated effects on neuromodulation are dependent on increased monoamine oxidase-A and acetylcholinesterase activity

**Figure 6:**
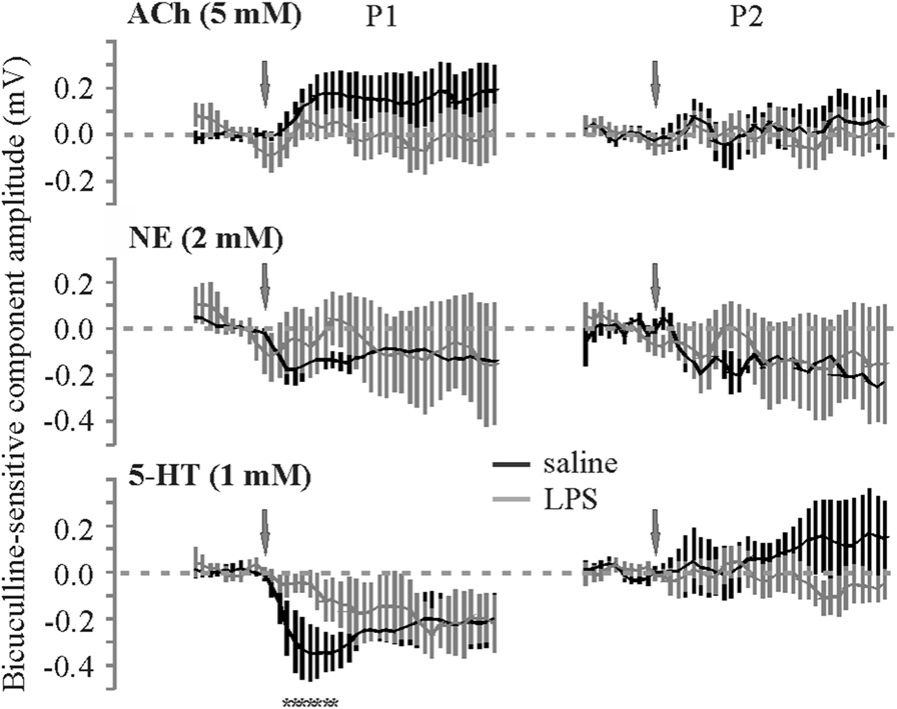
**Changes in GABA**_**A**_ **-mediated inhibition do not account for LPS-mediated changes in neuromodulator action.** Time series of the bicuculline-sensitive neuromodulator effects under control and LPS pretreatment conditions is plotted for comparison. Neuromodulator application in the presence of bicuculline (2 μm in perfusate) was subtracted from the time series of neuromodulator application before bicuculline administration (*N* = 5 slices from five animals; *P < 0.05, unpaired *t*-test). 5-HT, serotonin; ACh, acetylcholine; NE, norepinephrine; P1, pulse 1; P2, pulse 2; LPS, lipopolysaccharide.

Altered neuromodulation in LPS-treated brain slices is not due to an indirect reduction in inhibition. As we saw above, LPS treatment leads to a significant loss of both spontaneous and evoked inhibitory components (Figure 4). Given that neuromodulator effects are known to involve GABA_A_-mediated inhibition (Figure 2), we compared the bicuculline-sensitive component of the neuromodulator responses under saline or LPS pretreatment conditions (Figure 6). Each slice was administered, the neuromodulator twice, once before and once in the presence of bicuculline in the perfusate (saline-treated animals shown in Figure 2). To obtain the bicuculline-sensitive component of the neuromodulator response, we subtracted the response in bicuculline from the response immediately before bicuculline application. Although LPS-treated animals showed a small decrease in the bicuculline-sensitive component of the early 5-HT effect on the first pulse only, the bicuculline-sensitive components of neuromodulation were basically unaffected (Figure 6) suggesting that loss of inhibition in LPS-treated brain slices does not account for the alteration observed in neuromodulator effects.

**Figure 7:**
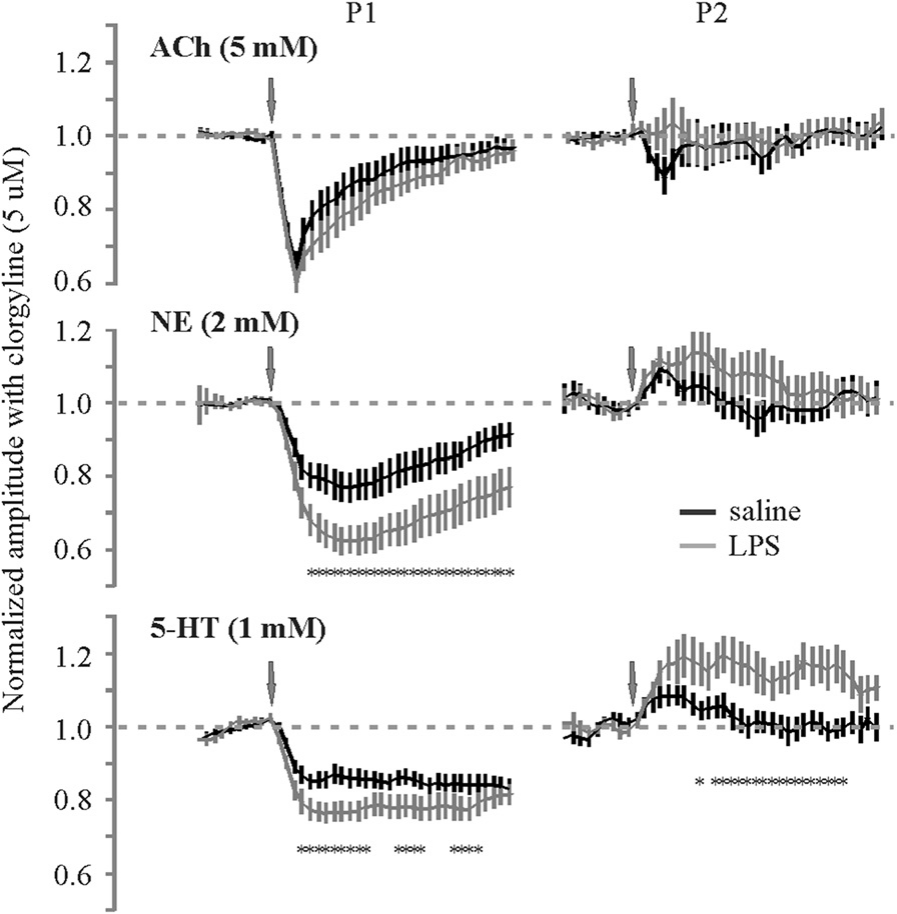
**Pharmacological inhibition of MAO-A shows increased NE- and 5-HT-mediated effects on the paired-pulse regimen.** Time series plots of pulse 1 and 2 amplitudes in the presence of the MAO-A inhibitor clorgyline illustrates an LPS-mediated enhancement of NE and 5-HT effects without altering ACh-mediated effects. (*N* = 18 to 24 slices from five animals in each group; *P < 0.05, unpaired *t*-test). 5-HT, serotonin; ACh, acetylcholine; NE, norepinephrine; LPS, lipopolysaccharide.

To evaluate whether changes in MAO-A activity is responsible for the LPS-mediated changes in the temporal profile of NE and 5-HT responses, we employed use of the MAO-A inhibitor clorgyline. To adequately block MAO-A activity in the perfused brain slice, slices were incubated in aCSF-containing clorgyline (5 μM) for 1 to 2 h prior to experimental recordings. As opposed to the reduced NE and 5-HT effects in LPS-treated slices in the absence of MAO-A block (Figure 5), we found significantly larger NE effects on pulse 1 and 5-HT effects on both pulse 1 and 2 of the paired-pulse regimen in slices from LPS-treated mice with no effect on ACh neuromodulation (Figure 7).

**Figure 8:**
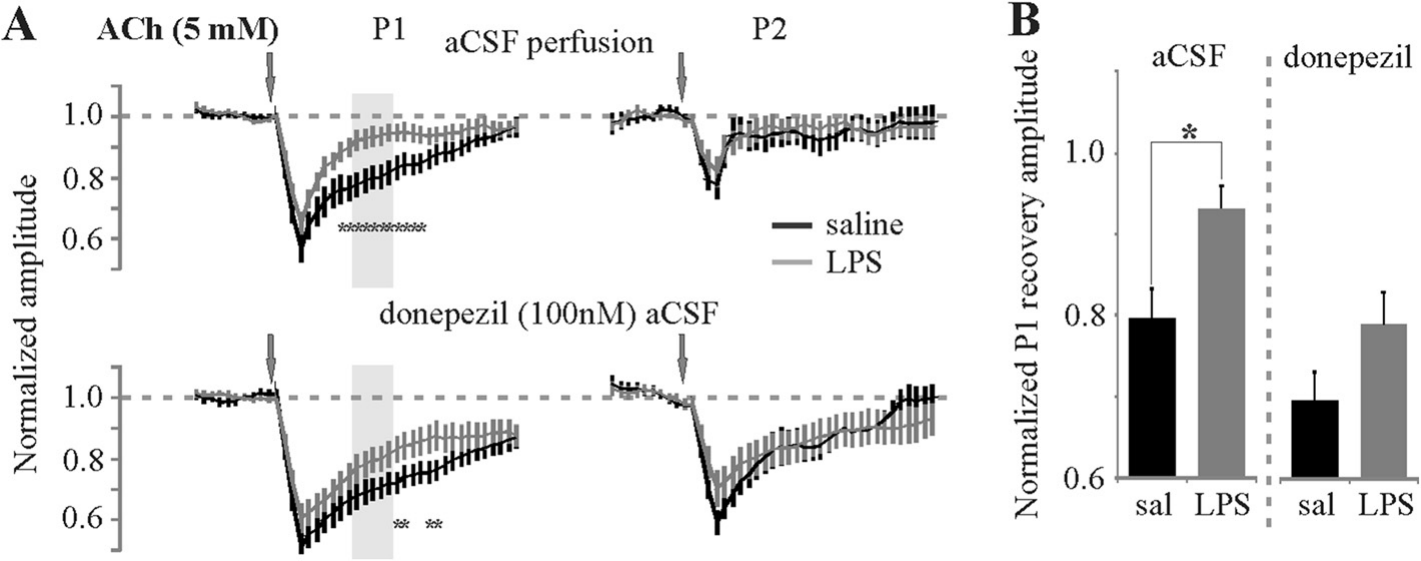
**Pharmacological inhibition of ACh-E blocks LPS-mediated increases in recovery of ACh-mediated effects. (A)** Time series plots of normalized pulse 1 and 2 amplitudes in the absence and presence of the ACh-E inhibitor donepezil illustrating the loss of an LPS-mediated enhancement in recovery of ACh-mediated effects (*P < 0.05, unpaired *t*-test). **(B)** Histograms comparing the normalized recovery of the first pulse as determined by the average of five stimulations demarcated by the grey-shaded boxes in **(A)** (*P < 0.05, Scheffé’s multiple comparison) (*N* = 14 slices from five animals in saline group, *N* = 15 slices from four animals in LPS group). ACh, acetylcholine; con, control; aCSF, artificial cerebral spinal fluid; don, donepezil; LPS, lipopolysaccharide; P1, pulse 1; P2, pulse 2.

We next evaluated the role of ACh-E on LPS-mediated changes in the temporal profile of ACh-mediated effects. To assess the role of ACh-E in the reduced duration of ACh effects in LPS-treated brain slices, we perfused the slice with the ACh-E inhibitor donepezil (100 nM). Unlike the effect of MAO-A inhibition on NE and 5-HT responses in LPS-treated slices, ACh did not show enhanced amplitude responses in the presence of donepezil (Figure 8A). Donepezil did, however, show a significant treatment effect on the recovery of P1 amplitude to ACh application (Figure 8B), supporting the notion of an increased ACh-E activity in mediating the LPS effects on ACh neuromodulation.

To further validate the role of MAO-A and ACh-E in the corresponding changes of neuromodulator temporal dynamics with LPS pretreatment, we assayed total MAO activity and ACh-E activity in hemi-brain homogenates from a subset of saline- (*n* = 14) and LPS- (*n* = 15) treated mice. Both total MAO (saline 1.00 ± 0.028 vs LPS 1.15 ± 0.034%; *P* = 0.0016, unpaired *t*-test) and ACh-E (saline 1.00 ± 0.027 vs LPS 1.23 ± 0.043%; *P* = 0.0001, unpaired *t*-test) activities were significantly increased in LPS-treated mice.

## Discussion

These studies use paired-pulse extracellular recordings with GABA_A_ pharmacology in superficial cortical layers as a means of assessing inflammatory effects on both inhibitory networks (Figure 1) and transient neuromodulation (Figure 2) of cortical networks in adult brain slices. Prior studies assessing neuromodulator effects on synaptic activity administer the different neuromodulators in the slice perfusate to allow the concentration to reach equilibrium throughout the tissue, resembling tonic neuromodulator action associated with general arousal and behavioural state. The present study employs bolus application of the neuromodulators upstream of the recording electrode in the slice perfusate to more accurately simulate phasic release of neuromodulators known to be associated with salient environmental stimuli that are necessary for transient modulation of executive functions. Few studies have ever assessed neuromodulator function on network activity in this way. Our results show that systemic LPS injection not only reduces CNS inhibitory networks (Figure 4) but also alters the temporal dynamics of neuromodulator activity (Figure 5) by increasing MAO-A (Figure 7) and ACh-E (Figure 8) activity. It should be noted that effects on temporal dynamics would not have been observed had we been using bath perfusion of the different neuromodulator concentrations or used neuromodulator concentrations insufficient to saturate local catabolic enzymes.

### Neuromodulator effects on somatosensory cortical networks

Using extracellular field recordings of a paired-pulse regimen, we were able to confirm some, but not all, of the previously reported effects of ACh, NE and 5-HT on spontaneous and evoked inhibition in patch-clamped cortical neurons.

In the bicuculline study of ACh, we were unable to uncover a GABA_A_-sensitive effect on either pulse 1 or 2 peak amplitude responses. This is directly in contrast with patch-clamp studies of principal layer II neurons in rat auditory [32] or entorhinal cortices [33], where they found an ACh-mediated decrease in evoked and an increase in spontaneous inhibition. As we observed very similar changes in P1 and P2 amplitude using both low and high concentrations, it is very possible that we were using a saturating concentration where the direct effects of ACh on cortical neurons overwhelms and/or masks any bicuculline-sensitive component. Alternatively, the low concentration of bicuculline used to avoid slice hyperexcitability may have only provided a fractional inhibition of the GABA_A_-mediated component making it too small to detect with extracellular field recordings. Although no bicuculline sensitivity was observed on peak ACh-mediated changes in amplitude, we found a non-significant trend for slower and incomplete recovery of pulse 1 amplitude in the presence of bicuculline. This may be attributed to a bicuculline-mediated lowering of the threshold for synapses to undergo cholinergic-mediated long-term depression (LTD) [25]. Kirkwood *et al*. [25] used a very similar paired-pulse model but with bath application of neuromodulator for 10 min to induce an LTD. In our study, bolus ACh application (transient <1 min) did not induce LTD under baseline conditions. However, with the increased excitability after addition of bicuculline, it is possible that subsequent ACh application might reach the threshold for LTD to occur.

Bicuculline-sensitive components of the NE effects on pulse 1 and 2 are consistent with an increase in both spontaneous and evoked inhibition, respectively. Reduction of pulse 1 inhibition in the presence of bicuculline correlates with an NE-mediated increase in spontaneous inhibition, whereas the reduced pulse 2 inhibition in bicuculline correlates with an NE-mediated increase in evoked inhibition. This is consistent with the previously shown α_1_-adrenergic receptor-mediated increase in frequency and amplitude of spontaneous inhibitory postsynaptic currents recorded (patch-clamp) from principal neurons in layer II/III of rat entorhinal cortex [34] and a β- and α_2_-adrenergic receptor-mediated increase in evoked inhibitory postsynaptic currents recorded from principal neurons in layer II/III of rat auditory cortex [35,36]. Thus, in contrast to bicuculline studies with ACh, use of bicuculline pharmacology does corroborate NE effects on inhibitory network activity under the conditions used in these studies.

Studies assessing bicuculline sensitivity of 5-HT effects on the paired-pulse regimen demonstrate a strong GABA_A_ component involved in the reduction of the first pulse which is consistent with previous studies showing 5-HT_1A_ receptor involvement in 5-HT effects in rat entorhinal cortex [37,38]. However, in contrast to the decrease in evoked inhibition observed in a previous study in the entorhinal cortex [38], we did not observe any impact on evoked inhibition. Lack of an observed effect on evoked inhibition may be explained similarly to lack of responses seen with ACh listed above. The slower onset of depression with 5-HT may suggest a role for GABA_B_ receptors and/or an effect on longer-term plasticity. In any case, 5-HT-mediated shaping of cortical networks shows the greatest dependence on recruitment of GABA_A_ receptors.

### Systemic LPS reduces cortical inhibition

The observed loss of spontaneous and evoked inhibition in these studies supports an elevation of inflammatory cytokines. Systemic LPS can increase inflammatory cytokines IL-1β [6], IL-6 [7] and TNF-α [6] in the brain. The measured increase in nitrates/nitrites as an indirect measure of nitric oxide in response to LPS in these studies suggests an inflammatory-mediated increase in nitric oxide synthase activity, supporting the probability of increased inflammatory cytokine levels. As TNF-α has been shown to cause internalization of GABA_A_ receptors [17] and IL-6 has been shown to reduce GABA_A_ receptor-mediated inhibition [14], the loss of cortical inhibition observed in these studies is consistent with an LPS-mediated elevation in the neuroinflammatory state.

### LPS-mediated effects on neuromodulation are dependent on increased monoamine oxidase-A and acetylcholinesterase activity

Given that all three neuromodulators used in this study have been shown to exert their neuromodulatory action partially through changes in inhibitory GABAergic mechanisms, it is possible that alteration in neuromodulator function under inflammatory conditions could be due to the loss of inhibitory network function. We found that although all neuromodulators showed a concentration-dependent increase in temporal recovery of responses, only 5-HT showed some loss of the bicuculline-sensitive neuromodulator effect in LPS-treated animal slices. This strongly suggested that mechanisms other than loss of inhibition are involved in the observed LPS-mediated changes in neuromodulator function.

Use of selective MAO-A and ACh-E inhibitors as well as activity measures from hemi-brain homogenates support the notion that changes in enzyme activity in slices from LPS-treated mice account for the altered temporal kinetics of the observed neuromodulator actions. Use of the ACh-E inhibitor donepezil, directly inhibited the significant increase in ACh effect recovery observed in slices from LPS-treated mice. MAO-A blockade results, however, proved more complex. Given the fact that noradrenergic and serotonergic receptor densities are adaptable to changes in the environment, it is conceivable that receptor densities were increased in LPS-treated mice to account for reduced NE and 5-HT concentrations as a result of increased MAO-A activity. Thus, the enhanced NE and 5-HT responses in slices from LPS-treated mice in the presence of MAO-A block may be indirect evidence of an enhanced MAO-A activity. The observed effects of pharmacological inhibition in slices is consistent with the observed increases in total MAO and ACh-E activity found in LPS-treated brain homogenates.

MAO-A expression has been widely shown to increase in response to chronic stress [18] and glucocorticoids [19,20] whereas ACh-E expression has been shown to increase in response to IL-1 [22] as well as oxidative stress [23,24]. Thus, similar to mechanisms for the loss of the observed inhibition, increased MAO-A and ACh-E activity are consistent with an increased inflammatory cytokine expression in brain slices from mice pretreated with LPS. Altered neuromodulator actions may be involved in the cognitive and emotional aspects of LPS-mediated sickness behaviour as well as provide a link to long-term neuropsychiatric and neurodegenerative disorders.

## Conclusions

These studies use a novel and simple extracellular recording paradigm combined with bicuculline pharmacology and transient bolus application of neuromodulators to evaluate cortical inhibitory networks and neuromodulator-mediated changes in integrated cortical network activity. We use this model to show that LPS-mediated neuroinflammation affects neuromodulation through effects on both cortical inhibition and increases in MAO-A and ACh-E activity. Although results only show an impact on the temporal dynamics of neuromodulator action under conditions used in these studies, it can be envisioned that an increase in MAO-A and ACh-E activity will also reduce neuromodulator action on a spatial scale. Thus, inflammatory-mediated sickness behaviour may involve changes in inhibition as well as loss of sensitivity to the different neuromodulators known to be important in determining behavioural state.

Given the significant role of neuromodulators in behavioural state and cognitive processes, it is possible that an inflammatory-mediated change in neuromodulator action plays a role in LPS-induced sickness behaviour and could help define the link between infection and neuropsychiatric/degenerative conditions.
